# Clinicopathological Characteristics and Prognosis of HER2-Low Early-Stage Breast Cancer: A Single-Institution Experience

**DOI:** 10.3389/fonc.2022.906011

**Published:** 2022-06-16

**Authors:** Hangcheng Xu, Yiqun Han, Yun Wu, Yan Wang, Qing Li, Pin Zhang, Peng Yuan, Yang Luo, Ying Fan, Shanshan Chen, Ruigang Cai, Qiao Li, Fei Ma, Binghe Xu, Jiayu Wang

**Affiliations:** ^1^ Department of Medical Oncology, National Cancer Center/National Clinical Research Center for Cancer/Cancer Hospital, Chinese Academy of Medical Sciences and Peking Union Medical College, Beijing, China; ^2^ Department of VIP Medical Services, National Cancer Center/National Clinical Research Center for Cancer/Cancer Hospital, Chinese Academy of Medical Sciences and Peking Union Medical College, Beijing, China

**Keywords:** HER2-low, HER2-zero, breast cancer, prognosis, landmark analysis

## Abstract

**Background:**

Human epidermal growth factor 2 (HER2)-low breast cancer, which is defined as HER2 1+ or 2+ in immunohistochemistry without gene amplification, accounts for a considerable part of all breast cancers. However, it remains controversial whether HER2-low breast cancer is a distinct entity. Our aim was to compare the clinicopathological features and survival outcomes between HER2-zero and HER2-low early breast cancer.

**Methods:**

The study was a retrospective analysis that enrolled 1,039 patients with available HER2 expression data in a single institute from 2013 to 2014, of whom 262 HER2-positive patients were excluded from the subsequent analysis. The remaining patients were divided into HER2-zero and HER2-low groups. Each group was further categorized into a hormone receptor (HR)-positive and an HR-negative subgroup. Clinicopathological characteristics were collected and compared between HER2-zero and HER2-low groups. The primary endpoint was disease-free survival (DFS) and overall survival (OS), which were analyzed using the Kaplan–Meier method with log-rank test, landmark analysis, and Cox proportional hazards model.

**Results:**

A total of 777 non-HER2-positive patients were included in this analysis, of whom 126, 552, 53, and 46 patients were HR-positive/HER2-zero, HR-positive/HER2-low, HR-negative/HER2-zero, and HR-negative/HER2-low, respectively. No significant difference in DFS and OS was detected between the HER2-zero group and the HER2-low group when paired by HR status. Landmark analysis with a time point set at 5 years indicated that HR-positive/HER2-low patients had a better DFS compared with HR-positive/HER2-zero patients after 5 years (*p* = 0.0047). HER2-low status was an independent prognostic factor for DFS after 5 years [hazard ratio (HR) 0.31, 95% confidence interval (CI) 0.13–0.75, *p* = 0.01].

**Conclusion:**

The clinicopathological characteristics and prognosis of HER2-zero and HER2-low breast cancer were similar regardless of HR status. Patients with HR-positive/HER2-low tumors tended to have a better DFS than their HR-positive/HER2-zero counterparts after 5 years.

## Introduction

According to the latest global cancer burden statistics, breast cancer is currently the most frequently diagnosed malignancy in women (1), which is regarded as a curable disease in the early stage. Traditionally, breast cancer is divided into four different subtypes according to the positivity or negativity of hormone receptor (HR) and human epidermal growth factor 2 (HER2), namely, HR+/HER2-, HR+/HER2+, HR-/HER2+, and HR-/HER2-. Distinct molecular classification leads to the heterogeneity of breast cancer, and the patient-tailored treatment based on it is widely used in clinical practice (2, 3). HER2-positive breast cancer accounts for 15%–20% of the entire breast cancer and is related to poor prognosis (4). The introduction of HER2-targeted therapy comprising monoclonal antibodies, tyrosine kinase inhibitors (TKIs), and antibody–drug conjugates (ADCs) dramatically improved the outcomes of patients with HER2-positive tumors (5).

At present, the estimation of HER2 status is conducted on the basis of the American Society of Clinical Oncology (ASCO)/College of American Pathologist (CAP) guidelines (6), using immunohistochemistry (IHC) and/or *in situ* hybridization (ISH). The guidelines defined HER2 IHC 3+ or IHC 2+ with ISH+ as HER2-positive and HER2 IHC 1+ or IHC 2+ with ISH- as HER2-negative. It is observed that there also exists HER2 expression in HER2-negative tumors, but in smaller levels (HER2-low status), which might be different from HER2-zero and HER2-positive breast cancer in treatment pattern and prognosis.

Until now, the definition of HER2-low status is varied across different studies without formal nomenclature. Earlier studies usually defined the “moderate HER2 expression” as HER2 IHC 2+/ISH- (7, 8), while most of the latest studies also categorize HER2 IHC 1+ as HER2-low status (9). It is estimated that HER2-low breast cancer occupies about 45%–55% of all breast cancers (9). With the emergence of trastuzumab duocarmazine (10) and trastuzumab deruxtecan (11), as well as the implementation of their clinical trials, HER2-low breast cancer has attracted more and more attention. Recent studies implied that HER2-low breast cancer might be a distinct kind with different gene profiles (12) and biological characteristics (13). So far, the clinical implication of HER2-low breast cancer remains disputable. Several studies indicated that patients with HER2-low tumors experienced poorer survival compared with HER2-zero counterparts (7, 8, 14, 15), entailing more aggressive treatment. Other studies, however, showed that no significant difference was detected between HER2-zero and HER2-low breast cancer (12, 16–18). Also, there were studies revealing better survival of HER2-low breast cancer (13, 19). Given this, we conducted a retrospective analysis attempting to determine whether there exist differences between HER2-low and HER2-zero breast cancer.

## Methods

### Patients

This retrospective research enrolled patients with early breast cancer who had undergone surgical procedures in Cancer Hospital, Chinese Academy of Medical Sciences (CHCAMS) between January 2013 and December 2014. Newly diagnosed women with definite surgery records and confirmed pathological assessments were considered eligible. Patients without available HER2 status were excluded from the analysis. Clinicopathological features including age, menopausal status, histological type, grade, TNM stage, estrogen receptor (ER) status, progesterone receptor (PgR) status, Ki-67 index, topoisomerase IIα (TOP2A) expression, and adjuvant treatment (endocrine therapy, radiotherapy, and chemotherapy) were extracted from the medical records. This study received approval from the Ethics Committee of CHCAMS and was conducted in accordance with the Declaration of Helsinki. All the participants signed informed consent forms.

### Pathological Assessment

HER2 protein expression was determined by IHC and was stratified as IHC 0, IHC 1+, IHC 2+, and IHC 3+. Samples with IHC 2+ were subsequently subjected to fluorescence *in situ* hybridization (FISH) to detect the *HER2* gene amplification. Based on the outcomes of IHC and FISH, HER2 status was classified into three groups: HER2-zero (IHC 0), HER2-low (including IHC 1+ and IHC 2+/FISH-negative), and HER2-positive (IHC 2+/FISH-positive and IHC 3+). Tumors were regarded as ER- or PgR-positive if ER or PgR ≥ 1%. HR-positive was defined as ER- and/or PgR-positive while both ER- and PgR-negative were considered as HR-negative. All the procedures were performed according to the ASCO/CAP guidelines (6, 20). The initial pathological diagnosis of operative specimens was utilized for data analysis.

### Statistical Analysis

The baseline clinicopathological variables were presented as frequencies and percentages. The relation between clinicopathological characteristics and HER2 status was determined by chi-square test or Fisher’s exact test for categorical variables and by Mann–Whitney’s test for continuous variables. Disease-free survival (DFS) was defined as the time from the primary diagnosis to disease recurrence or death from any cause. Overall survival (OS) was defined as the interval from breast cancer diagnosis to death from any cause or the last follow-up. The Kaplan–Meier method was used to generate the survival curves. The difference in DFS and OS was accessed by log-rank test. In case that the scenario did not conform to the proportional hazards assumption, landmark analysis was conducted to assess survival outcomes within and after a designated time point, which was known as landmark time. The cutoff time point was chosen based on visual inspection of the Schoenfeld residual plots and Kaplan–Meier curves. The univariate and multivariate Cox proportional hazards model was employed to identify prognostic factors. The variables with Wald’s *p*-value < 0.2 in the univariate analysis were selected for subsequent multivariate analysis. A two-tailed *p*-value of less than 0.05 was considered statistically significant. All data analysis was performed using SPSS version 23.0 (SPSS, Chicago, IL, USA) and R software version 4.1.1 (http://www.r-project.org).

## Results

### Clinicopathological Features

**Figure 1:**
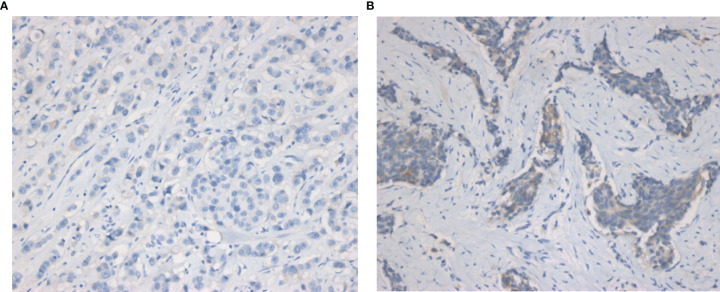
Representative IHC images of HER2-zero and HER2-low breast cancers. **(A)** IHC images of HER2-zero breast cancer. **(B)** IHC images of HER2-low breast cancer (magnification ×200 in each picture). IHC, immunochemistry.

A total of 1,091 patients diagnosed with early-stage breast cancer between January 2013 and December 2014 were identified, among which 52 HER2 IHC2+ patients with unknown FISH results and 262 HER2-positive patients were excluded. A total of 777 patients with HER2-zero or HER2-low tumors were eventually analyzed (**Figure 1**). The details are provided in **Supplementary Table 1**. In particular, the percentage of older patients (age ≥ 70) was lower in the HER2-low group (*p* = 0.02). As for the pathological characteristics, tumor grade tended to be advanced in the HER2-zero group (grade III, 35.20% vs. 18.39%, *p* < 0.001). The rate of HR positivity in the HER2-low group was significantly higher (92.31% vs. 70.39%, *p* < 0.001). The Ki-67 index and TOP2A expression seemed to be lower in the HER2-low group (Ki-67 ≤ 30%, 74.58% vs. 59.68%, *p* < 0.001; TOP2A ≤ 60%, 96.66% vs. 92.74%, *p* < 0.001). There was no statistical significance in the tumor size and lymph node status between these two groups. Regarding the treatment, more patients (85.12%) in the HER2-low group received endocrine therapy (*p* < 0.001) while no difference was detected in radiotherapy and chemotherapy.

The included patients were then divided according to HR status (**Table 1**). HR-positive and HR-negative patients accounted for 87.26% and 12.74%, respectively. In the HR-positive subgroup, HER2-low patients tended to be older (age ≥ 70) and have a higher PgR-positive rate compared with HER2-zero patients (*p* = 0.009 and *p* = 0.019, respectively). Other baseline characteristics were comparable between two subgroups. In the HR-negative subgroup, all features were similar in HER2-zero and HER2-low patients.

**Table 1:** Baseline characteristics stratified by HR status.

Characteristics	HR-positive *N* = 678				*p*-value	HR-negative *N* = 99				*p*-value
	HER2-zero		HER2-low			HER2-zero		HER2-low		
	*N* = 126		*N* = 552			*N* = 53		*N* = 46		
	No.	Percent (%)	No.	Percent (%)		No.	Percent (%)	No.	Percent (%)	
**Age, years**										
Median (range)	50 (30–88)		50 (24–86)		0.955	50 (33–81)		53 (28–75)		0.133
<40	15	11.9	68	12.32	**0.009**	7	13.21	5	10.87	0.513
40–49	47	37.3	199	36.05		19	35.85	10	21.74	
50–59	26	20.63	153	27.72		16	30.19	20	43.48	
60–69	22	17.46	107	19.38		7	13.21	8	17.39	
>=70	16	12.7	25	4.53		4	7.55	3	6.52	
**Menopausal status**					0.863					0.402
Premenopausal	70	55.56	302	54.71		24	45.28	17	36.96	
Postmenopausal	56	44.44	250	45.29		29	54.72	29	63.04	
**Histological type**					0.078					1
No special type	109	86.51	499	90.4		49	92.45	42	91.3	
Invasive lobular	7	5.56	11	1.99		0	0	0	0	
Other	10	7.94	42	7.61		4	7.55	4	8.7	
**Grade**					0.056					0.085
Grade I	14	11.11	78	14.13		0	1.75	1	2.17	
Grade II	72	57.14	359	65.04		16	29.82	12	26.09	
Grade III	26	20.63	81	14.67		37	68.42	29	63.04	
Unknown	14	11.11	34	6.16		0	0	4	8.7	
**T stage**					0.245					0.544
T0/is/1	86	68.25	346	62.68		31	58.49	24	52.17	
T2	35	27.78	193	34.96		20	37.74	19	41.3	
T3	3	2.38	9	1.63		2	3.77	1	2.17	
T4	1	0.79	1	0.18		0	0	2	4.35	
Tx	1	0.79	3	0.54		0	0	0	0	
**N stage**					0.428					0.094
N0	68	53.97	299	54.17		32	60.4	34	73.33	
N1	33	26.19	159	28.8		13	24.5	5	11.11	
N2	16	12.7	56	10.14		2	3.8	5	11.11	
N3	7	5.56	36	6.52		6	11.3	2	4.44	
Nx	2	1.59	2	0.36		0	0	0	0	
**Pathological stage**					0.371					0.911
I	50	39.68	230	41.67		21	39.62	19	40	
II	49	38.89	224	40.58		24	45.28	19	42.22	
III	24	19.05	94	17.03		8	15.09	8	17.78	
Unknown	3	2.38	4	0.72		0	0	0	0	
**ER status**					0.505					
Positive	118	93.65	525	95.11		-	-	-	-	
Negative	8	6.35	27	4.89		-	-	-	-	
**PgR status**					**0.019**					
Positive	114	90.48	528	95.65		-	-	-	-	
Negative	12	9.52	24	4.35		-	-	-	-	
**Ki-67**					0.468					0.218
<15%	42	33.33	178	32.25		2	3.77	4	8.7	
15%–30%	51	40.48	253	45.83		7	13.21	11	23.91	
>30%	33	26.19	121	21.92		44	83.02	31	67.39	
**TOP2A**					0.378					0.157
<30%	100	79.37	461	83.51		13	24.53	17	36.96	
30%–60%	22	17.46	82	14.86		31	58.49	18	39.13	
>60%	4	3.17	9	1.63		9	16.98	11	23.91	
**Adjuvant endocrine therapy**					0.119					0.85
Yes	108	85.71	505	91.49		3	5.66	4	8.7	
AIs	54	42.86	239	43.30		1	1.89	2	4.35	
Tamoxifen	52	41.27	236	42.75		2	3.77	2	4.35	
AIs/Tamoxifen	2	1.59	30	5.43		0	0	0	0	
No	13	10.32	31	5.62		49	92.45	41	89.13	
Unknown	5	3.97	16	2.9		1	1.89	1	2.17	
**Adjuvant radiotherapy**					0.464					0.608
Yes	49	38.89	244	44.2		23	54.72	20	43.48	
No	70	55.56	286	51.81		29	43.4	25	54.35	
Unknown	7	5.56	22	3.99		1	1.89	1	2.17	
**Adjuvant chemotherapy**					0.355					1
Yes	77	61.11	369	66.85		47	88.68	41	89.13	
Anthracycline	10	7.94	41	7.43		1	1.89	0	0	
Taxane	22	17.46	73	13.22		25	47.17	20	43.48	
Anthracycline + Taxane	45	35.71	255	46.2		21	39.62	21	45.65	
No	44	34.92	170	30.8		5	9.43	4	8.7	
Unknown	5	3.97	13	2.36		1	1.89	1	2.17	

HR, hormone receptor; HER2, human epiderma growth factor receptor 2; ER, estrogen receptor; PgR, progesterone receptor; TOP2A, topoisomerase II alpha; AIs, aromatase inhibitors. Bold values indicate statistically significant results.

### Prognosis Analysis

**Figure 2:**
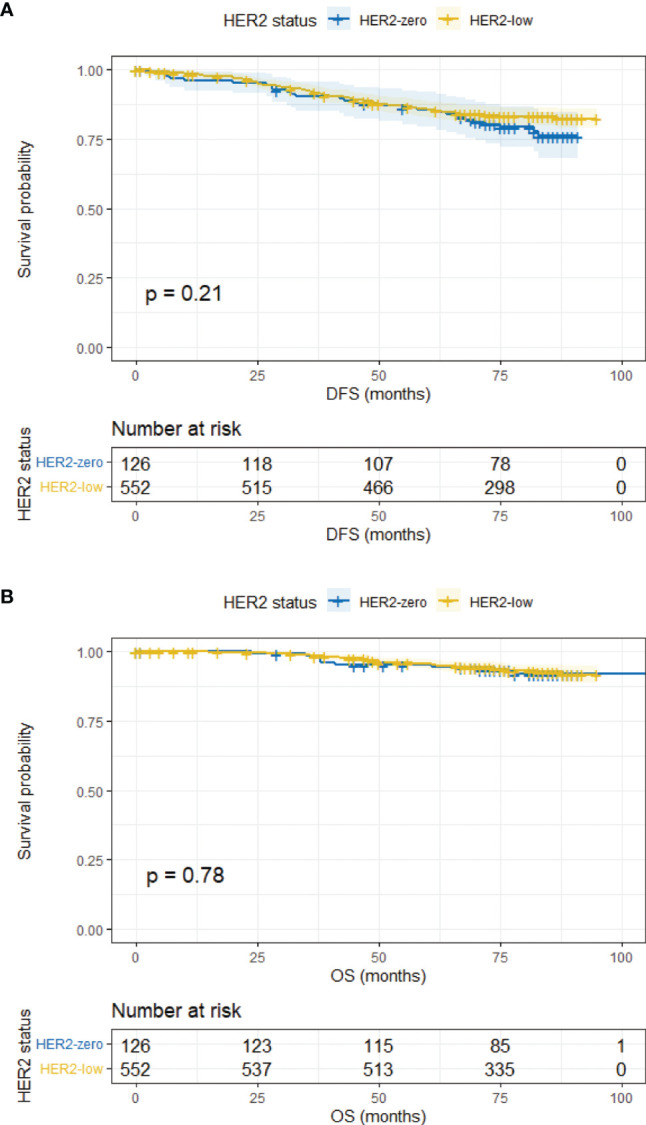
DFS and OS in HR-positive/HER2-zero and HR-positive/HER2-low patients. **(A)** DFS in HR-positive/HER2-zero and HR-positive/HER2-low patients. **(B)** OS in HR-positive/HER2-zero and HR-positive/HER2-low patients.

**Figure 3:**
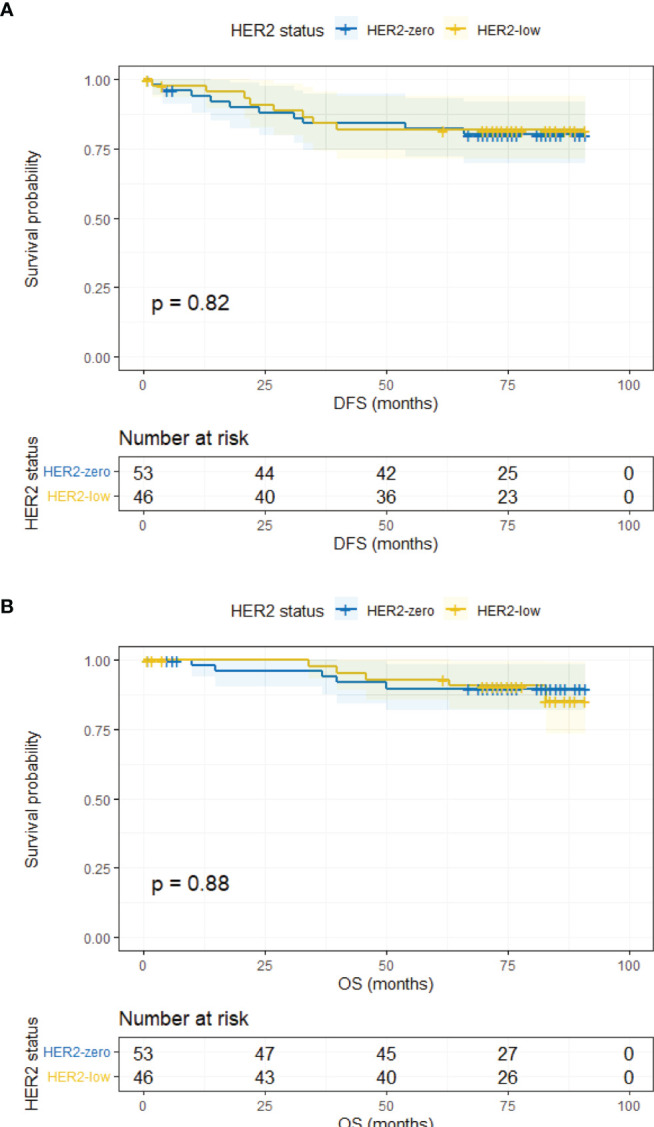
DFS and OS in HR-negative/HER2-zero and HR-negative/HER2-low patients. **(A)** DFS in HR-negative/HER2-zero and HR-negative/HER2-low patients. **(B)** OS in HR-negative/HER2-zero and HR-negative/HER2-low patients.

The median follow-up time was 78 months (95% CI, 76.43–79.57 months). In the overall population, HER2-zero and HER2-low patients had comparable DFS and OS (5-year DFS, 83.9% vs. 85.2%, *p* = 0.183; 5-year OS, 93.0% vs. 95.3%, *p* = 0.585) (**Supplementary Figure 1**). Survival outcomes were further analyzed according to HR status. In the HR-positive subgroup, the DFS and OS of HER2-low patients were similar to HER2-zero counterparts (5-year DFS, 85.5% vs. 84.6%, *p* = 0.206; 5-year OS, 95.5% vs. 94.3%, *p* = 0.776) (**Figure 2**). Analogous results were yielded in the HR-negative subgroup between the HER2-low and HER2-zero population (5-year DFS, 81.9% vs. 80.1%, *p* = 0.819; 5-year OS, 90.6% vs. 89.8%, *p* = 0.880) (**Figure 3**).

**Figure 4:**
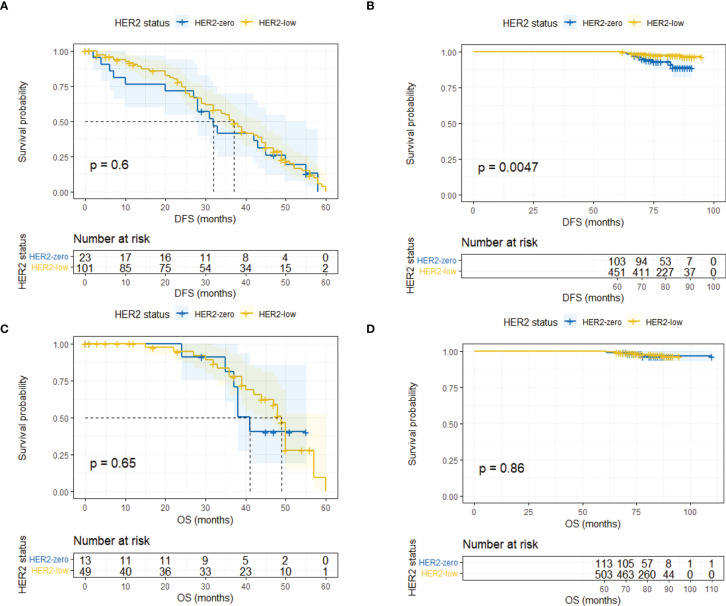
Landmark analysis of DFS and OS in HR-positive/HER2-zero and HR-positive/HER2-low patients. **(A)** DFS in HR-positive/HER2-zero and HR-positive/HER2-low patients within 60 months. **(B)** DFS in HR-positive/HER2-zero and HR-positive/HER2-low patients after 60 months. **(C)** OS in HR-positive/HER2-zero and HR-positive/HER2-low patients within 60 months. **(D)** OS in HR-positive/HER2-zero and HR-positive/HER2-low patients after 60 months.

**Figure 5:**
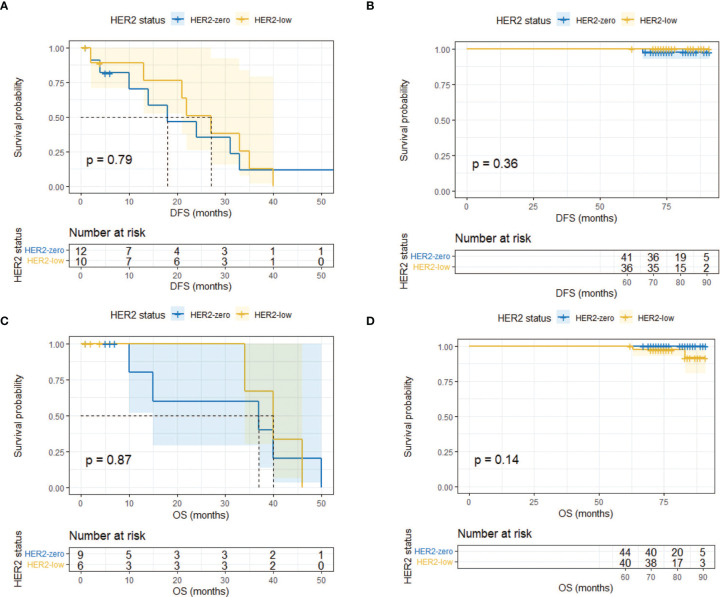
Landmark analysis of DFS and OS in HR-negative/HER2-zero and HR-negative/HER2-low patients. **(A)** DFS in HR-negative/HER2-zero and HR-negative/HER2-low patients within 60 months. **(B)** DFS in HR-negative/HER2-zero and HR-negative/HER2-low patients after 60 months. **(C)** OS in HR-negative/HER2-zero and HR-negative/HER2-low patients within 60 months. **(D)** OS in HR-negative/HER2-zero and HR-negative/HER2-low patients after 60 months.

Landmark analysis was introduced afterwards with a time point setting at 60 months. In the HR-positive cohort, HER2-low patients had a significantly better DFS compared with HER2-zero patients after 5 years (*p* = 0.0047) while the same outcome did not occur within 5 years (*p* = 0.604) (**Figures 4A, B**). No difference was observed regarding OS within and after 5 years (**Figures 4C, D**). As for the HR-negative cohort, two subgroups had a similar DFS and OS in the landmark analysis (**Figure 5**).

Lastly, we conducted univariate and multivariate analysis of prognosis in the overall population, the HR-positive subgroup, and the HR-negative subgroup. The results are summarized in **Supplementary Tables 2**, **3**, **4**. The Cox regression analysis in different time segments was also performed. In HR-positive patients, HER2-low status was a significant prognostic factor for a better DFS compared with HER-zero status after 5 years (HR 0.31, 95% CI 0.13–0.75, *p* = 0.01) (**Table 2**), while HER2 status did not display similar predictive effects for HR-positive patients in other cases (**Supplementary Tables 4**, **5**).

**Table 2:** Univariate and multivariate analysis of variables correlated with DFS in HR-positive patients within and after 5 years.

Variables	Univariate analysis				Multivariate analysis			
	DFS (within 5 years)		DFS (after 5 years)		DFS (within 5 years)		DFS (after 5 years)	
	Hazard Ratio (95% CI)	*p*-Value	Hazard Ratio (95% CI)	*p*-Value	Hazard Ratio (95% CI)	*p*-Value	Hazard Ratio (95% CI)	*p*-Value
**Age, years**		0.06		0.189		0.061		0.344
<40	Reference		Reference		Reference		Reference	
40–49	0.51 (0.27–0.98)	0.043	0.23 (0.05–1.03)	0.054	0.55 (0.27–1.14)	0.11	0.20 (0.04–0.93)	0.04
50–59	0.38 (0.20–0.73)	0.004	0.58 (0.16–2.16)	0.416	0.33 (0.16–0.69)	0.003	2.43 (0.04–1.70)	0.154
60–69	0.53 (0.26–1.10)	0.087	0.91 (0.26–3.24)	0.888	0.49 (0.21–1.13)	0.093	0.25 (0.03–2.05)	0.197
>=70	0.35 (0.12–1.00)	0.05	1.37 (0.31–6.12)	0.683	0.48 (0.14–1.64)	0.241	0.25 (0.03–2.35)	0.224
**Menopausal status**		0.648		0.045				0.128
Premenopausal	Reference		Reference				Reference	
Postmenopausal	0.91 (0.60–1.37)	0.648	2.53 (1.02–6.28)	0.045			3.82 (0.68–21.47)	0.128
**Histological type**		0.096				0.029		
No special type	Reference				Reference			
Invasive lobular	2.57 (0.79–8.37)	0.116			12.80 (1.50–109.02)	0.02		
Other	0.56 (0.25–1.23)	0.149			1.03 (0.37–2.84)	0.959		
**Grade**		0.072		0.463		0.016		
Grade I	Reference		Reference		Reference			
Grade II	0.59 (0.27–1.31)	0.193	3.24 (0.43–24.60)	0.257	0.65 (0.26–1.63)	0.358		
Grade III	1.10 (0.47–2.59)	0.826	4.99 (0.58–42.74)	0.142	1.54 (0.55–4.32)	0.417		
Unknown	0.60 (0.20–1.80)	0.36	1.94 (0.12–31.08)	0.639	0.25 (0.04–1.54)	0.134		
**T stage**		0.841		0.435				
T0/is/1	Reference		Reference					
T2	1.08 (0.71–1.65)	0.721	1.15 (0.46–2.88)	0.768				
T3	0.74 (0.32–1.73)	0.484	7.57 (0.99–58.00)	0.052				
T4	1.38 (0.19–10.06)	0.751	/	0.991				
**N stage**		0.008		0.371		< 0.001		
N0	Reference		Reference		Reference			
N1	1.53 (0.91–2.58)	0.11	1.06 (0.36–3.11)	0.912	1.97 (1.10–3.53)	0.023		
N2	1.08 (0.59–1.98)	0.8	2.86 (0.89–9.12)	0.077	1.14 (0.56–2.33)	0.722		
N3	2.58 (1.40–4.75)	0.002	2.58 (0.57–11.80)	0.221	4.17 (2.05–8.46)	< 0.001		
**HER2 status**		0.613		0.008				0.01
HER2-zero	Reference		Reference				Reference	
HER2-low	0.875 (0.52–1.47)	0.613	0.31 (0.13–0.73)	0.008			0.31 (0.13–0.75)	0.01
**Ki-67**		0.13		0.294		0.032		
<15%	Reference		Reference		Reference			
15%–30%	1.15 (0.71–1.88)	0.575	2.47 (0.80–7.69)	0.118	1.67 (0.93–3.01)	0.087		
>30%	1.75 (0.99–3.10)	0.055	2.00 (0.53–7.46)	0.305	2.64 (1.28–5.45)	0.009		
**TOP2A**		0.162				0.2		
<30%	Reference				Reference			
30%–60%	1.50 (0.90–2.49)	0.123			0.92 (0.49–1.73)	0.803		
>60%	0.34 (0.05–2.46)	0.284			0.14 (0.02–1.21)	0.074		
**Endocrine therapy**		0.621		0.028				0.085
No/Unknown	Reference		Reference				Reference	
Yes	0.87 (0.50–1.51)	0.621	0.30 (0.10–0.88)	0.028			0.37 (0.12–1.15)	0.085
**Radiotherapy**		0.496		0.64				
No/Unknown	Reference		Reference					
Yes	1.15 (0.77–1.74)	0.496	1.23 (0.52–2.89)	0.64				
**Chemotherapy**		0.298		0.267				
No/Unknown	Reference		Reference					
Yes	1.27 (0.81–1.98)	0.298	1.77 (0.65–4.83)	0.267				

DFS, disease-free survival; HR, hormone receptor; HER2, human epiderma growth factor receptor 2; TOP2A, topoisomerase II alpha.

## Discussion

Breast cancer has always been at the forefront of precision therapy; one of the representative examples is the targeted therapy for HER2-positive breast cancer. The advent of novel ADCs makes it possible for patients with moderate HER2 expression, a rather huge population, to benefit from anti-HER2 therapy (9). Nevertheless, there is an ongoing debate on whether HER2-low breast cancer is a distinct entity, but there is a lack of data on this subject, particularly in a Chinese population. A recent published study by our institution analyzed the HER2-low breast cancer in the metastatic setting and concluded that HER2 low expression was related to a better OS (21). In the present study, we focused on early-stage patients, comparing the clinicopathological characteristics and prognosis between HER2-zero and HER2-low breast cancer with a different HR status. In particular, we found that HER2-low breast cancer tended to have a better DFS compared with HER2-zero breast cancer in the HR-positive cohort in the long term.

In this retrospective analysis, 1,039 patients with early-stage breast cancer had available HER2 status, 57.56% of whom had HER2-low breast cancer. The proportion was in line with previous studies by and large (9). The demographics, pathology, and treatment patterns within the 777 non-HER2-positive breast cancer patients were compared. A prominent feature of breast cancer with low HER2 expression was a sizeable increase in the positive rate of HR compared with HER2-zero counterparts (92.31% vs. 70.39%, *p* < 0.001), which was relatively higher than other reported data (64.0%–90.2%) (12, 13, 16, 22). At the genetic level, recent data showed that HER2-low breast cancers had a higher expression of luminal-related genes compared with HER2-zero tumors (12). Additionally, in our study, other pathological features such as tumor grade and Ki-67 index were different between HER2-zero and HER2-low groups, the differences of which disappeared when the overall population is further divided according to HR status. In the HR-positive population, there were fewer older patients (age ≥ 70, 4.53% vs. 12.70%) and more PgR-positive patients (95.65% vs. 90.48%) in the HER2-low subgroup, while in the HR-negative population, all the recorded features were balanced in the HER2-zero and HER2-low subgroups. We speculated that the significant imbalance in HR positivity resulted in the difference between HER2-zero and HER2-low baseline characteristics. Many other studies also discussed patients with a different HER2 status in a distinguished HR background (13, 16), and the clinicopathological features of HER2-low breast cancer are not fully elucidated so far. A newly published research based on Chinese women with breast cancer (*n* = 523) implied that the HER2-low expression was associated with distinct clinical and molecular features (such as lower Ki-67 expression and particular types of gene mutation) (23). Our present study did not support the idea that HER2-low breast cancer was different from HER2-zero regarding biology. Whether HER2-low breast cancer harbors more aggressive features remains unclear in view of the mixed results from different studies (12, 13, 24).

The prognosis of HER2-low breast cancer remains disputable at present. We analyzed the DFS and OS in the HER2-zero and HER2-low entities, both in the overall population and in the HR-positive/negative subgroups. However, no significant differences in DFS and OS were detected between patients with HER2-zero or HER2-low tumors regardless of HR status. The outcomes were consistent with a study from Japan (16). However, newly published data from Korea showed that breast cancer−specific survival (BCSS) instead of OS was better in HER2-low entity in spite of the HR status (24). Another multicenter study demonstrated that HER2-low breast cancer showed both better relapse−free survival (RFS) and OS in a non-metastatic setting (25). The relatively small sample size and the short follow-up time contributed to the differences between their studies and ours. Furthermore, we found that the Kaplan–Meier curves between two groups crossed each other, which indicated that it might violate the assumption for the proportional hazards model. Thus, we performed landmark analyses with the designated time point at 60 months. Results signified that the HER2-low group had a better DFS than the HER2-zero group in HR-positive patients after 60 months (*p* = 0.0047), while no marked difference was detected within 60 months (*p* = 0.604). We also performed Cox regression in divided time intervals to determine whether HER-low expression could predict the prognosis. Results showed that HER2-low expression was an independent prognostic factor for DFS after 5 years with a reduced risk of 69% compared with HER2-zero expression (HR 0.31, 95% CI 0.13–0.75, *p* = 0.01). We did not obtain similar outcomes in HR-positive patients regarding OS. The same scenario like ours could also been seen in another study that showed that the disparity in DFS widened after 5 years between HER2-zero and HER2-low groups in HR-positive patients (26). Likewise, a recent large-scale study indicated that better RFS of HER2-low patients emerged in the HR-positive subgroup with longer follow-up after 6 years (25). Although it did not reach statistical significance, Horisawa et al. found that HER2-low patients (*n* = 3,169) tended to have a better prognosis than those in the HER2-zero group (*n* = 838) regardless of HR status, and it seemed that the better prognosis of the HER2-low population became increasingly apparent as time went on (16).

Notably, the better prognosis of HER2-low tumor only presented in the HR-positive patients based on current evidence. One previous study by Mutai et al. also compared the prognosis between the HER2-zero and HER2-low group in early-stage luminal disease and found that HER2-low expression was related to a better OS, DFS, and distant disease-free survival (DDFS) in women with high genomic risk (19). A recent study illustrated the PAM50 intrinsic subtype profiles of HER2-low tumors and concluded that the gene expression between HER2-zero and HER2-low tumors was dramatically different within HR-positive disease while no difference was detected in TNBC, justifying that HR-positive/HER2-low tumors were a more distinct biological entity than HR-negative/HER2-low tumors (12). Another study utilizing similar methods also emphasized the significance of considering HR status in the HER2-low classification (26). It was still unclear why HR-positive/HER2-low breast cancer patients had a better DFS in the long run. The following explanations might partly account for it. On the one hand, compared with HER2-zero tumors, HER2-low tumors consisted of more HER2-enriched subtypes (26), which was a predictive factor for prognosis (27). The possible reduced aggressiveness and other intricate unknown biology of HER2-low breast cancer might also be attributed to its better prognosis (24). Previous exploratory analyses from the phase III ExteNET trial (NCT00878709) showed the greater efficacy of neratinib, an irreversible pan-HER TKI, in the HR-positive population (28). The elaborate cross-talk between HR and HER2 was believed to play a role in it (29). We speculated that a similar mechanism also applied to HER2-low tumors since they also had some extent of HER2 expression (30), but undoubtedly more research into it was warranted. Of note, we utilized the primary pathological diagnosis in this analysis, while HER2 status is actually dynamic. Two similar studies showed that the HER2-low expression evolved from primary to recurrent breast cancer (30) and between early and advanced-stage breast cancer (31), which indicated that a second biopsy might be necessary during the disease progression for suitable treatment options.

So far, ADCs showed a promising future in treating HER2-low breast cancer due to their particular structures (32). Take trastuzumab deruxtecan (T-DXd, DS-8201a) as an example; a phase Ib study that enrolled 54 patients with heavily pretreated HER2-low metastatic breast cancer (MBC) showed a median progression-free survival (PFS) of 11.1 months (11). The latest data presented at the 2021 San Antonio Breast Cancer Symposium (SABCS) disclosed the results of DAISY, a phase II study that assessed the activity of T-DXd in advanced breast cancer (ABC) with different extents of HER2 expression. The study further confirmed the efficacy of T-DXd in HER2-low MBC with a best overall response (BOR) of 37.5% (27/72) and a median PFS of 6.7 months (33). Other ADCs such as trastuzumab duocarmazine (SYD985) (10) and RC 48-ADC (34) also proved to be effective in treating HER2-low breast cancer. Obviously, the clinical trials evaluating the efficacy of these drugs were primarily designed for MBC. In view of the success in MBC, the investigators also evaluated the necessity of advancing treatment with novel ADCs to early-stage breast cancer. However, based on the current state of knowledge, the present outcome did not support the notion.

Several limitations should be considered when interpreting the results. To begin with, this was a retrospective analysis with some potential bias due to its nature. Secondly, the sample size was relatively small especially for the HR-negative population. HER2 IHC2+ patients without available FISH results were excluded from this analysis, which might lead to patient selection bias to some extent. In addition, the IHC evaluation of HER2 was short of a central pathological review and results might vary from person to person. Moreover, it was difficult to fully distinguish HER2-zero tumors from HER2 IHC 1+ tumors using the current detection techniques (35), which could impair the credibility of our research. Despite these limitations, we reported the clinicopathological features and survival outcome of HER2-low entity and hoped that it could deepen the understanding of HER2-low breast cancer.

## Conclusion

In conclusion, our study showed that the clinicopathological characteristics between HER2-zero and HER2-low breast cancer were basically consistent in HR-positive or HR-negative settings, respectively. Landmark analysis indicated that patients with HR-positive/HER2-low tumors had a superior DFS compared with their HR-positive/HER2-zero counterparts after 5 years.

## Data Availability Statement

The original contributions presented in the study are included in the article/**Supplementary Material**. Further inquiries can be directed to the corresponding author.

## Ethics Statement

The studies involving human participants were reviewed and approved by the Ethics Committee of Cancer Hospital, Chinese Academy of Medical Sciences (CHCAMS). The patients/participants provided their written informed consent to participate in this study.

## Author Contributions

JW conceived the study. All authors collected data. HX, YH, and YuW analyzed data. HX wrote the manuscript. All authors contributed to the article and approved the submitted version.

## Conflict of Interest

The authors declare that the research was conducted in the absence of any commercial or financial relationships that could be construed as a potential conflict of interest.

## Publisher’s Note

All claims expressed in this article are solely those of the authors and do not necessarily represent those of their affiliated organizations, or those of the publisher, the editors and the reviewers. Any product that may be evaluated in this article, or claim that may be made by its manufacturer, is not guaranteed or endorsed by the publisher.

## References

[1] Sung H Ferlay J Siegel RL Laversanne M Soerjomataram I Jemal A. Global Cancer Statistics 2020: GLOBOCAN Estimates of Incidence and Mortality Worldwide for 36 Cancers in 185 Countries. CA: Cancer J Clin (2021) 71(3):209–49. doi: 10.3322/caac.21660 33538338

[2] Perou CM Sørlie T Eisen MB van de Rijn M Jeffrey SS Rees CA. Molecular Portraits of Human Breast Tumours. Nature (2000) 406(6797):747–52. doi: 10.1038/35021093 10963602

[3] Desmedt C Haibe-Kains B Wirapati P Buyse M Larsimont D Bontempi G. Biological Processes Associated With Breast Cancer Clinical Outcome Depend on the Molecular Subtypes. Clin Cancer Res (2008) 14(16):5158–65. doi: 10.1158/1078-0432.Ccr-07-4756 18698033

[4] Slamon DJ Clark GM Wong SG Levin WJ Ullrich A McGuire WL. Human Breast Cancer: Correlation of Relapse and Survival With Amplification of the HER-2/Neu Oncogene. Sci (New York NY) (1987) 235(4785):177–82. doi: 10.1126/science.3798106 3798106

[5] Cesca MG Vian L Cristóvão-Ferreira S Pondé N de Azambuja E. HER2-Positive Advanced Breast Cancer Treatment in 2020. Cancer Treat Rev (2020) 88:102033. doi: 10.1016/j.ctrv.2020.102033 32534233

[6] Wolff AC Hammond MEH Allison KH Harvey BE Mangu PB Bartlett JMS. Human Epidermal Growth Factor Receptor 2 Testing in Breast Cancer: American Society of Clinical Oncology/College of American Pathologists Clinical Practice Guideline Focused Update. J Clin Oncol (2018) 36(20):2105–22. doi: 10.1200/jco.2018.77.8738 29846122

[7] Ignatov T Eggemann H Burger E Fettke F Costa SD Ignatov A. Moderate Level of HER2 Expression and its Prognostic Significance in Breast Cancer With Intermediate Grade. Breast Cancer Res Treat (2015) 151(2):357–64. doi: 10.1007/s10549-015-3407-2 25926338

[8] Eggemann H Ignatov T Burger E Kantelhardt EJ Fettke F Thomssen C. Moderate HER2 Expression as a Prognostic Factor in Hormone Receptor Positive Breast Cancer. Endocrine Relat Cancer (2015) 22(5):725–33. doi: 10.1530/erc-15-0335 26187126

[9] Tarantino P Hamilton E Tolaney SM Cortes J Morganti S Ferraro E. HER2-Low Breast Cancer: Pathological and Clinical Landscape. J Clin Oncol (2020) 38(17):1951–62. doi: 10.1200/jco.19.02488 32330069

[10] Banerji U van Herpen CML Saura C Thistlethwaite F Lord S Moreno V. Trastuzumab Duocarmazine in Locally Advanced and Metastatic Solid Tumours and HER2-Expressing Breast Cancer: A Phase 1 Dose-Escalation and Dose-Expansion Study. Lancet Oncol (2019) 20(8):1124–35. doi: 10.1016/s1470-2045(19)30328-6 31257177

[11] Modi S Park H Murthy RK Iwata H Tamura K Tsurutani J. Antitumor Activity and Safety of Trastuzumab Deruxtecan in Patients With HER2-Low-Expressing Advanced Breast Cancer: Results From a Phase Ib Study. J Clin Oncol (2020) 38(17):1887–96. doi: 10.1200/jco.19.02318 PMC7280051 32058843

[12] Schettini F Chic N Brasó-Maristany F Paré L Pascual T Conte B. Clinical, Pathological, and PAM50 Gene Expression Features of HER2-Low Breast Cancer. NPJ Breast Cancer (2021) 7(1):1. doi: 10.1038/s41523-020-00208-2 33397968 PMC7782714

[13] Denkert C Seither F Schneeweiss A Link T Blohmer JU Just M. Clinical and Molecular Characteristics of HER2-Low-Positive Breast Cancer: Pooled Analysis of Individual Patient Data From Four Prospective, Neoadjuvant Clinical Trials. Lancet Oncol (2021) 22(8):1151–61. doi: 10.1016/s1470-2045(21)00301-6 34252375

[14] Gilcrease MZ Woodward WA Nicolas MM Corley LJ Fuller GN Esteva FJ. Even Low-Level HER2 Expression May Be Associated With Worse Outcome in Node-Positive Breast Cancer. Am J Surg Pathol (2009) 33(5):759–67. doi: 10.1097/PAS.0b013e31819437f9 PMC3063383 19252432

[15] Camp RL Dolled-Filhart M King BL Rimm DL. Quantitative Analysis of Breast Cancer Tissue Microarrays Shows That Both High and Normal Levels of HER2 Expression Are Associated With Poor Outcome. Cancer Res (2003) 63(7):1445–8. 12670887

[16] Horisawa N Adachi Y Takatsuka D Nozawa K Endo Y Ozaki Y. The Frequency of Low HER2 Expression in Breast Cancer and a Comparison of Prognosis Between Patients With HER2-Low and HER2-Negative Breast Cancer by HR Status. Breast Cancer (Tokyo Japan) (2021) 29(2):234–41. doi: 10.1007/s12282-021-01303-3 34622383

[17] Gampenrieder SP Rinnerthaler G Tinchon C Petzer A Balic M Heibl S. Landscape of HER2-Low Metastatic Breast Cancer (MBC): Results From the Austrian AGMT_MBC-Registry. Breast Cancer Res: BCR (2021) 23(1):112. doi: 10.1186/s13058-021-01492-x 34906198 PMC8670265

[18] Hein A Hartkopf AD Emons J Lux MP Volz B Taran FA. Prognostic Effect of Low-Level HER2 Expression in Patients With Clinically Negative HER2 Status. Eur J Cancer (Oxf Engl: 1990) (2021) 155:1–12. doi: 10.1016/j.ejca.2021.06.033 34311211

[19] Mutai R Barkan T Moore A Sarfaty M Shochat T Yerushalmi R. Prognostic Impact of HER2-Low Expression in Hormone Receptor Positive Early Breast Cancer. Breast (Edinburgh Scotland) (2021) 60:62–9. doi: 10.1016/j.breast.2021.08.016 PMC8414540 34481367

[20] Hammond ME Hayes DF Dowsett M Allred DC Hagerty KL Badve S. American Society of Clinical Oncology/College Of American Pathologists Guideline Recommendations for Immunohistochemical Testing of Estrogen and Progesterone Receptors in Breast Cancer. J Clin Oncol (2010) 28(16):2784–95. doi: 10.1200/jco.2009.25.6529 PMC2881855 20404251

[21] Li Y Abudureheiyimu N Mo H Guan X Lin S Wang Z. In Real Life, Low-Level HER2 Expression May Be Associated With Better Outcome in HER2-Negative Breast Cancer: A Study of the National Cancer Center, China. Front Oncol (2021) 11:774577. doi: 10.3389/fonc.2021.774577 35111669 PMC8801428

[22] de Moura Leite L Cesca MG Tavares MC Santana DM Saldanha EF Guimarães PT. HER2-Low Status and Response to Neoadjuvant Chemotherapy in HER2 Negative Early Breast Cancer. Breast Cancer Res Treat (2021) 190(1):155–63. doi: 10.1007/s10549-021-06365-7 34409551

[23] Zhang G Ren C Li C Wang Y Chen B Wen L. Distinct Clinical and Somatic Mutational Features of Breast Tumors With High-, Low-, or non-Expressing Human Epidermal Growth Factor Receptor 2 Status. BMC Med (2022) 20(1):142. doi: 10.1186/s12916-022-02346-9 35484593 PMC9052533

[24] Won HS Ahn J Kim Y Kim JS Song JY Kim HK. Clinical Significance of HER2-Low Expression in Early Breast Cancer: A Nationwide Study From the Korean Breast Cancer Society. Breast Cancer Res: BCR (2022) 24(1):22. doi: 10.1186/s13058-022-01519-x 35307014 PMC8935777

[25] Tan R Ong WS Lee KH Lim AH Park S Park YH. HER2 Expression, Copy Number Variation and Survival Outcomes in HER2-Low non-Metastatic Breast Cancer: An International Multicentre Cohort Study and TCGA-METABRIC Analysis. BMC Med (2022) 20(1):105. doi: 10.1186/s12916-022-02284-6 35296300 PMC8928638

[26] Agostinetto E Rediti M Fimereli D Debien V Piccart M Aftimos P. HER2-Low Breast Cancer: Molecular Characteristics and Prognosis. Cancers (2021) 13(11):2824. doi: 10.3390/cancers13112824 34198891 PMC8201345

[27] Prat A Pascual T De Angelis C Gutierrez C Llombart-Cussac A Wang T. HER2-Enriched Subtype and ERBB2 Expression in HER2-Positive Breast Cancer Treated With Dual HER2 Blockade. J Natl Cancer Inst (2020) 112(1):46–54. doi: 10.1093/jnci/djz042 31037288 PMC7850037

[28] Chan A Moy B Mansi J Ejlertsen B Holmes FA Chia S. Final Efficacy Results of Neratinib in HER2-Positive Hormone Receptor-Positive Early-Stage Breast Cancer From the Phase III ExteNET Trial. Clin Breast Cancer (2021) 21(1):80–91.e7. doi: 10.1016/j.clbc.2020.09.014 33183970

[29] Johnston SR. New Strategies in Estrogen Receptor-Positive Breast Cancer. Clin Cancer Res (2010) 16(7):1979–87. doi: 10.1158/1078-0432.Ccr-09-1823 20332324

[30] Miglietta F Griguolo G Bottosso M Giarratano T Lo Mele M Fassan M. Evolution of HER2-Low Expression From Primary to Recurrent Breast Cancer. NPJ Breast Cancer (2021) 7(1):137. doi: 10.1038/s41523-021-00343-4 34642348 PMC8511010

[31] Tarantino P Gandini S Nicolò E Trillo P Giugliano F Zagami P. Evolution of Low HER2 Expression Between Early and Advanced-Stage Breast Cancer. Eur J Cancer (2022) 163:35–43. doi: 10.1016/j.ejca.2021.12.022 35032815

[32] Pondé N Aftimos P Piccart M. Antibody-Drug Conjugates in Breast Cancer: A Comprehensive Review. Curr Treat Opt Oncol (2019) 20(5):37. doi: 10.1007/s11864-019-0633-6 30931493

[33] Diéras V Deluche E Lusque A Pistilli B Bachelot T Pierga J-Y. Trastuzumab Deruxtecan (T-DXd) for Advanced Breast Cancer Patients (ABC), Regardless HER2 Status: A Phase II Study With Biomarkers Analysis (DAISY). SABCS; San Antonio Texas 78229 USA (2021). In Proceedings of the 2021 San Antonio Breast Cancer Symposium; December 7-10, 2021; San Antonio, TX, United States. Abstract PD8-02.

[34] Wang J Liu Y Zhang Q Feng J Fang J Chen X. RC48-ADC, a HER2-Targeting Antibody-Drug Conjugate, in Patients With HER2-Positive and HER2-Low Expressing Advanced or Metastatic Breast Cancer: A Pooled Analysis of Two Studies. J Clin Oncol (2021) 39:1022. doi: 10.1200/JCO.2021.39.15_suppl.1022

[35] Marchiò C Annaratone L Marques A Casorzo L Berrino E Sapino A. Evolving Concepts in HER2 Evaluation in Breast Cancer: Heterogeneity, HER2-Low Carcinomas and Beyond. Semin Cancer Biol (2021) 72:123–35. doi: 10.1016/j.semcancer.2020.02.016 32112814

