# Supplementary material for: Clinicopathological Characteristics and Prognosis of HER2-Low Early-Stage Breast Cancer: A Single-Institution Experience
Source: Front Oncol. 2022 Jun 16;12:906011. doi: 10.3389/fonc.2022.906011 (PMC9245921; doi:10.3389/fonc.2022.906011)

**Supplementary Materials**

**Supplementary Table 1** Baseline characteristics comparison between HER2-zero and HER2-low breast cancer patients.

| Characteristics | HER2 status | | | | P-value |
| --- | --- | --- | --- | --- | --- |
|  | **HER2-zero (N=179)** | | **HER2-low (N=598)** | |  |
|  | No. | Percent (%) | No. | Percent (%) |  |
| **Age, years** |  |  |  |  |  |
| Median (range) | 50 (30-88) |  | 50 (24-86) |  | 0.921 |
| <40 | 22 | 12.29 | 73 | 12.21 | **0.02** |
| 40-49 | 66 | 36.87 | 209 | 34.95 |  |
| 50-59 | 42 | 23.46 | 173 | 28.93 |  |
| 60-69 | 29 | 16.2 | 115 | 19.23 |  |
| ≥70 | 20 | 11.17 | 28 | 4.68 |  |
| **Menopausal status** |  |  |  |  | 0.845 |
| Premenopausal | 94 | 52.51 | 319 | 53.34 |  |
| Postmenopausal | 85 | 47.49 | 279 | 46.66 |  |
| **Histological type** |  |  |  |  | 0.269 |
| No special type | 158 | 88.27 | 541 | 90.47 |  |
| Invasive lobular | 7 | 3.91 | 11 | 1.84 |  |
| Other | 14 | 7.82 | 46 | 7.69 |  |
| **Grade** |  |  |  |  | **<0.001** |
| Grade I | 14 | 7.82 | 79 | 13.21 |  |
| Grade II | 88 | 49.16 | 371 | 62.04 |  |
| Grade III | 63 | 35.2 | 110 | 18.39 |  |
| Unknown | 14 | 7.82 | 38 | 6.35 |  |
| **T stage** |  |  |  |  | 0.583 |
| T0/is/1 | 117 | 65.36 | 370 | 61.87 |  |
| T2 | 55 | 30.73 | 212 | 35.45 |  |
| T3 | 5 | 2.79 | 10 | 1.67 |  |
| T4 | 1 | 0.56 | 3 | 0.5 |  |
| Unknown | 1 | 0.56 | 3 | 0.5 |  |
| **N stage** |  |  |  |  | 0.692 |
| N0 | 100 | 55.87 | 333 | 55.69 |  |
| N1 | 46 | 25.7 | 164 | 27.42 |  |
| N2 | 18 | 10.06 | 61 | 10.2 |  |
| N3 | 13 | 7.26 | 38 | 6.35 |  |
| Unknown | 2 | 1.12 | 2 | 0.33 |  |
| **Pathological stage** |  |  |  |  | 0.652 |
| I | 71 | 39.66 | 249 | 41.64 |  |
| II | 73 | 40.78 | 243 | 40.64 |  |
| III | 32 | 17.88 | 102 | 17.06 |  |
| Unknown | 3 | 1.68 | 4 | 0.67 |  |
| **ER status** |  |  |  |  | **<0.001** |
| Positive | 118 | 65.92 | 525 | 87.79 |  |
| Negative | 61 | 34.08 | 73 | 12.21 |  |
| **PgR status** |  |  |  |  | **<0.001** |
| Positive | 114 | 63.69 | 528 | 88.29 |  |
| Negative | 65 | 36.31 | 70 | 11.71 |  |
| **HR status** |  |  |  |  | **<0.001** |
| Positive | 126 | 70.39 | 552 | 92.31 |  |
| Negative | 53 | 29.61 | 46 | 7.69 |  |
| **Ki-67** |  |  |  |  | **<0.001** |
| <15% | 44 | 24.58 | 182 | 30.43 |  |
| 15%-30% | 58 | 32.4 | 264 | 44.15 |  |
| >30% | 77 | 43.02 | 152 | 25.42 |  |
| **TOP2A** |  |  |  |  | **<0.001** |
| <30% | 113 | 63.13 | 478 | 79.93 |  |
| 30%-60% | 53 | 29.61 | 100 | 16.72 |  |
| >60% | 13 | 7.26 | 20 | 3.34 |  |
| **Adjuvant endocrine therapy** |  |  |  |  | **<0.001** |
| Yes | 111 | 62.01 | 509 | 85.12 |  |
| Aromatase inhibitor | 55 | 30.73 | 241 | 40.3 |  |
| Tamoxifen | 54 | 30.17 | 238 | 39.8 |  |
| Aromatase inhibitor/Tamoxifen | 2 | 1.12 | 30 | 5.02 |  |
| No | 62 | 34.64 | 72 | 12.04 |  |
| Unknown | 6 | 3.35 | 17 | 2.84 |  |
| **Adjuvant radiotherapy** |  |  |  |  | 0.942 |
| Yes | 78 | 43.58 | 264 | 44.15 |  |
| No | 93 | 51.96 | 311 | 52.01 |  |
| Unknown | 8 | 4.47 | 23 | 3.85 |  |
| **Adjuvant chemotherapy** |  |  |  |  | 0.73 |
| Yes | 124 | 69.27 | 410 | 68.56 |  |
| Anthracycline | 11 | 6.15 | 41 | 6.86 |  |
| Taxane | 47 | 26.26 | 93 | 15.55 |  |
| Anthracycline + Taxane | 66 | 36.87 | 276 | 46.15 |  |
| No | 49 | 27.37 | 174 | 29.1 |  |
| Unknown | 6 | 3.35 | 14 | 2.34 |  |

*HER2* human epiderma growth factor receptor 2, *ER* estrogen receptor, *PR* progesterone receptor, *HR* hormone receptor, *TOP2A* topoisomerase II alpha

**Supplementary Table 2** Univariate and multivariate analysis of variables correlated with DFS and OS in the whole population.

| Variables | Univariate analysis | | | | Multivariate analysis | | | |
| --- | --- | --- | --- | --- | --- | --- | --- | --- |
|  | DFS | | OS | | DFS | | OS | |
|  | Hazard Ratio (95%CI) | *P* Value | Hazard Ratio (95%CI) | *P* Value | Hazard Ratio (95%CI) | *P* Value | Hazard Ratio (95%CI) | *P* Value |
| **Age, years** |  | 0.094 |  | 0.009 |  | 0.68 |  | 0.433 |
| <40 | Reference |  | Reference |  | Reference |  | Reference |  |
| 40-49 | 0.57 (0.33-0.99) | 0.047 | 0.52 (0.21-1.27) | 0.15 | 0.67 (0.38-1.18) | 0.163 | 0.75 (0.30-1.87) | 0.537 |
| 50-59 | 0.99 (0.59-1.68) | 0.973 | 1.07 (0.47-2.44) | 0.875 | 0.81 (0.44-1.52) | 0.518 | 1.11 (0.47-2.58) | 0.817 |
| 60-69 | 0.74 (0.41-1.34) | 0.323 | 0.57 (0.21-1.58) | 0.281 | 0.71 (0.33-1.51) | 0.373 | 0.77 (0.27-2.21) | 0.625 |
| >=70 | 1.07 (0.51-2.23) | 0.856 | 2.28 (0.88-5.92) | 0.089 | 0.67 (0.27-1.69) | 0.399 | 1.86 (0.62-5.54) | 0.269 |
| **Menopausal status** |  | 0.043 |  | 0.359 |  | 0.354 |  |  |
| Premenopausal | Reference |  | Reference |  | Reference |  |  |  |
| Postmenopausal | 1.42 (1.01-1.99) | 0.043 | 1.28 (0.76-2.18) | 0.359 | 1.27 (0.77-2.11) | 0.354 |  |  |
| **Histological type** |  | 0.719 |  | 0.778 |  |  |  |  |
| No special type | Reference |  | Reference |  |  |  |  |  |
| Invasive lobular | 0.94 (0.30-2.95) | 0.915 | 1.66 (0.40-6.83) | 0.482 |  |  |  |  |
| Other | 0.75 (0.36-1.52) | 0.419 | 0.97 (0.35-2.69) | 0.955 |  |  |  |  |
| **Grade** |  | 0.01 |  | 0.032 |  | 0.021 |  | 0.266 |
| Grade I | Reference |  | Reference |  | Reference |  | Reference |  |
| Grade II | 2.11 (1.02-4.36) | 0.044 | 6.63 (0.90-48.53) | 0.063 | 1.81 (0.85-3.86) | 0.126 | 4.64 (0.60-35.80) | 0.141 |
| Grade III | 3.20 (1.50-6.82) | 0.003 | 11.88 (1.59-88.53) | 0.016 | 3.16 (1.33-7.50) | 0.009 | 7.43 (0.88-63.06) | 0.066 |
| Unknown | 1.65 (0.60-4.54) | 0.336 | 5.82 (0.61-55.95) | 0.127 | 1.05 (0.37-2.99) | 0.931 | 4.89 (0.49-49.15) | 0.177 |
| **T stage** |  | <0.001 |  | 0.002 |  | 0.038 |  | 0.709 |
| T0/is/1 | Reference |  | Reference |  | Reference |  | Reference |  |
| T2 | 1.36 (0.95-1.94) | 0.095 | 1.79 (1.03-3.11) | 0.04 | 0.99 (0.67-1.45) | 0.95 | 1.02 (0.57-1.83) | 0.956 |
| T3 | 5.55 (2.67-11.54) | <0.001 | 4.91 (1.48-16.21) | 0.009 | 3.59 (1.59-8.12) | 0.002 | 1.87 (0.50-7.05 | 0.354 |
| T4 | 4.02 (0.99-16.37) | 0.053 | 11.38 (2.69-48.05) | 0.001 | 1.72 (0.38-7.72) | 0.481 | 2.84 (0.52-15.48) | 0.228 |
| **N stage** |  | <0.001 |  | <0.001 |  | <0.001 |  | <0.001 |
| N0 | Reference |  | Reference |  | Reference |  | Reference |  |
| N1 | 1.78 (1.16-2.75) | 0.009 | 3.46 (1.57-7.63) | 0.002 | 1.87 (1.18-2.98) | 0.008 | 3.66 (1.59-8.43) | 0.002 |
| N2 | 3.42 (2.10-5.57) | <0.001 | 7.86 (3.44-17.92) | <0.001 | 4.01 (2.20-7.33) | <0.001 | 7.47 (2.72-20.50) | <0.001 |
| N3 | 6.76 (4.15-11.01) | <0.001 | 17.79 (8.07-39.22) | <0.001 | 7.46 (4.05-13.75) | <0.001 | 16.87 (6.17-46.11) | <0.001 |
| **ER status** |  | 0.812 |  | 0.139 |  |  |  | 0.38 |
| Negative | Reference |  | Reference |  |  |  | Reference |  |
| Positive | 0.95 (0.61-1.48) | 0.812 | 0.63 (0.34-1.16) | 0.139 |  |  | 1.70 (0.52-5.59) | 0.38 |
| **PgR status** |  | 0.007 |  | 0.027 |  | 0.141 |  | 0.303 |
| Negative | Reference |  | Reference |  | Reference |  | Reference |  |
| Positive | 0.58 (0.39-0.86) | 0.007 | 0.51 (0.28-0.93) | 0.027 | 0.65 (0.37-1.15) | 0.141 | 0.60 (0.23-1.59) | 0.303 |
| **HER2 status** |  | 0.185 |  | 0.586 |  | 0.857 |  |  |
| HER2-zero | Reference |  | Reference |  | Reference |  |  |  |
| HER2-low | 0.77 (0.53-1.13) | 0.185 | 0.85 (0.46-1.55) | 0.586 | 0.96 (0.63-1.47) | 0.857 |  |  |
| **Ki-67** |  | 0.164 |  | 0.091 |  | 0.032 |  | 0.477 |
| <15% | Reference |  | Reference |  | Reference |  | Reference |  |
| 15%-30% | 1.50 (0.97-2.30) | 0.065 | 1.80 (0.86-3.76) | 0.12 | 1.46 (0.93-2.30) | 0.099 | 1.62 (0.74-3.55) | 0.232 |
| >30% | 1.43 (0.90-2.29) | 0.13 | 2.32 (1.09-4.93) | 0.029 | 0.81 (0.46-1.43) | 0.47 | 1.31 (0.55-3.15) | 0.545 |
| **TOP2A** |  | 0.212 |  | 0.256 |  |  |  |  |
| <30% | Reference |  | Reference |  |  |  |  |  |
| 30%-60% | 1.38 (0.93-2.05) | 0.108 | 1.54 (0.85-2.79) | 0.157 |  |  |  |  |
| >60% | 0.75 (0.28-2.05) | 0.58 | 0.48 (0.07-3.48) | 0.465 |  |  |  |  |
| **Endocrine therapy** |  | 0.011 |  | 0.004 |  | 0.633 |  | 0.241 |
| No/Unknown | Reference |  | Reference |  | Reference |  | Reference |  |
| Yes | 0.61 (0.41-0.89) | 0.011 | 0.44 (0.25-0.77) | 0.004 | 0.87 (0.49-1.54) | 0.633 | 0.57 (0.23-1.46) | 0.241 |
| **Radiotherapy** |  | 0.02 |  | 0.004 |  | 0.284 |  | 0.91 |
| No/Unknown | Reference |  | Reference |  | Reference |  | Reference |  |
| Yes | 1.50 (1.07-2.10) | 0.02 | 2.25 (1.30-3.90) | 0.004 | 0.79 (0.52-1.22) | 0.284 | 0.96 (0.47-1.96) | 0.91 |
| **Chemotherapy** |  | 0.112 |  | 0.136 |  | 0.166 |  | 0.359 |
| No/Unknown | Reference |  | Reference |  | Reference |  | Reference |  |
| Yes | 1.37 (0.93-2.02) | 0.112 | 1.63 (0.86-3.09) | 0.136 | 0.72 (0.45-1.15) | 0.166 | 0.69 (0.31-1.52) | 0.359 |

*DFS* Disease-free survival, *OS* Overall survival, *ER* estrogen receptor, *PgR* progesterone receptor, *HER2* human epiderma growth factor receptor 2, *TOP2A* topoisomerase II alpha

**Supplementary Table 3** Univariate and multivariate analysis of variables correlated with DFS and OS in HR-positive patients.

| Variables | Univariate analysis | | | | Multivariate analysis | | | |
| --- | --- | --- | --- | --- | --- | --- | --- | --- |
|  | DFS | | OS | | DFS | | OS | |
|  | Hazard Ratio (95%CI) | *P* Value | Hazard Ratio (95%CI) | *P* Value | Hazard Ratio (95%CI) | *P* Value | Hazard Ratio (95%CI) | *P* Value |
| **Age, years** |  | 0.125 |  | 0.105 |  | 0.421 |  | 0.197 |
| <40 | Reference |  | Reference |  | Reference |  |  |  |
| 40-49 | 0.53 (0.30-0.96) | 0.034 | 0.45 (0.18-1.13) | 0.088 | 0.61 (0.33-1.10) | 0.099 | 0.60 (0.23-1.54) | 0.288 |
| 50-59 | 0.96 (0.55-1.67) | 0.871 | 0.81 (0.34-1.94) | 0.642 | 0.80 (0.39-1.62) | 0.527 | 0.81 (0.32-2.04) | 0.655 |
| 60-69 | 0.73 (0.39-1.35) | 0.315 | 0.47 (0.16-1.36) | 0.164 | 0.60 (0.26-1.41) | 0.241 | 0.48 (0.15-1.52) | 0.213 |
| >=70 | 0.82 (0.36-1.88) | 0.634 | 1.49 (0.52-4.29) | 0.461 | 0.57 (0.19-1.65) | 0.295 | 1.81 (0.55-5.96) | 0.327 |
| **Menopausal status** |  | 0.107 |  | 0.598 |  | 0.331 |  |  |
| Premenopausal | Reference |  | Reference |  | Reference |  |  |  |
| Postmenopausal | 1.35 (0.94-1.94) | 0.107 | 1.17 (0.65-2.10) | 0.598 | 1.34 (0.74-2.40) | 0.331 |  |  |
| **Histological type** |  | 0.754 |  | 0.696 |  |  |  |  |
| No special type | Reference |  | Reference |  |  |  |  |  |
| Invasive lobular | 0.96 (0.30-3.02) | 0.941 | 1.80 (0.43-7.44) | 0.418 |  |  |  |  |
| Other | 0.75 (0.35-1.60) | 0.453 | 0.88 (0.27-2.84) | 0.827 |  |  |  |  |
| **Grade** |  | 0.017 |  | 0.048 |  | 0.064 |  | 0.26 |
| Grade I | Reference |  | Reference |  | Reference |  | Reference |  |
| Grade II | 2.11 (1.02-4.37) | 0.045 | 6.30 (0.86-46.28) | 0.071 | 1.82 (0.86-3.89) | 0.12 | 3.85 (0.51-29.27) | 0.193 |
| Grade III | 3.35 (1.52-7.37) | 0.003 | 12.04 (1.58-92.07) | 0.016 | 3.06 (1.28-7.33) | 0.012 | 6.63 (0.79-55.53) | 0.081 |
| Unknown | 1.74 (0.63-4.79) | 0.287 | 6.11 (0.64-58.73) | 0.117 | 1.36 (0.49-3.81) | 0.553 | 5.05 (0.51-50.02) | 0.166 |
| **T stage** |  | <0.001 |  | 0.008 |  | 0.056 |  | 0.824 |
| T0/is/1 | Reference |  | Reference |  | Reference |  | Reference |  |
| T2 | 1.34 (0.91-1.96) | 0.138 | 1.74 (0.94-3.22) | 0.076 | 1.01 (0.67-1.51) | 0.979 | 0.99 (0.52-1.88) | 0.963 |
| T3 | 5.64 (2.58-12.31) | <0.001 | 5.67 (1.70-18.93) | 0.005 | 3.57 (1.54-8.29) | 0.003 | 2.08 (0.58-7.47) | 0.26 |
| T4 | 4.03 (0.56-29.08) | 0.167 | 12.15 (1.63-90.34) | 0.015 | 1.35 (0.17-10.94) | 0.776 | 1.62 (0.17-15.48) | 0.674 |
| **N stage** |  | <0.001 |  | <0.001 |  | <0.001 |  | <0.001 |
| N0 | Reference |  | Reference |  | Reference |  | Reference |  |
| N1 | 1.81 (1.14-2.87) | 0.011 | 4.03 (1.63-9.98) | 0.003 | 1.64 (1.00-2.69) | 0.052 | 3.37 (1.32-8.58) | 0.011 |
| N2 | 3.50 (2.09-5.87) | <0.001 | 8.95 (3.47-23.08) | <0.001 | 3.59 (1.91-6.74) | <0.001 | 6.78 (2.29-20.08) | 0.001 |
| N3 | 5.86 (3.38-10.15) | <0.001 | 19.90 (7.93-49.90) | <0.001 | 5.14 (2.64-9.99) | <0.001 | 16.20 (5.56-47.17) | <0.001 |
| **HER2 status** |  | 0.208 |  | 0.777 |  |  |  |  |
| HER2-zero | Reference |  | Reference |  |  |  |  |  |
| HER2-low | 0.76 (0.49-1.17) | 0.208 | 0.90 (0.43-1.87) | 0.777 |  |  |  |  |
| **Ki-67** |  | 0.267 |  | 0.06 |  |  |  | 0.728 |
| <15% | Reference |  | Reference |  |  |  | Reference |  |
| 15%-30% | 1.41 (0.91-2.18) | 0.123 | 1.45 (0.67-3.12) | 0.342 |  |  | 1.38 (0.61-3.10) | 0.435 |
| >30% | 1.40 (0.84-2.33) | 0.201 | 2.51 (1.14-5.54) | 0.023 |  |  | 1.17 (0.42-3.26) | 0.758 |
| **TOP2A** |  | 0.088 |  | 0.117 |  | 0.418 |  | 0.941 |
| <30% | Reference |  | Reference |  | Reference |  | Reference |  |
| 30%-60% | 1.58 (1.01-2.45) | 0.044 | 2.01 (1.04-3.89) | 0.039 | 0.98 (0.58-1.66) | 0.946 | 1.16 (0.50-2.71) | 0.728 |
| **Endocrine therapy** |  | <0.001 |  | <0.001 |  | 0.005 |  | 0.008 |
| No/Unknown | Reference |  | Reference |  | Reference |  | Reference |  |
| Yes | 0.39 (0.24-0.64) | <0.001 | 0.27 (0.14-0.55) | <0.001 | 0.46 (0.27-0.79) | 0.005 | 0.32 (0.14-0.74) | 0.008 |
| **Radiotherapy** |  | 0.139 |  | 0.011 |  | 0.405 |  | 0.666 |
| No/Unknown | Reference |  | Reference |  | Reference |  | Reference |  |
| Yes | 1.31 (0.92-1.89) | 0.139 | 2.18 (1.19-3.99) | 0.011 | 0.82 (0.52-1.30) | 0.405 | 1.19 (0.55-2.58) | 0.666 |
| **Chemotherapy** |  | 0.092 |  | 0.282 |  | 0.453 |  |  |
| No/Unknown | Reference |  | Reference |  | Reference |  |  |  |
| Yes | 1.42 (0.94-2.13) | 0.092 | 1.44 (0.74-2.78) | 0.282 | 0.83 (0.50-1.36) | 0.453 |  |  |

*HR* hormone receptor, *DFS* Disease-free survival, *OS* Overall survival, *HER2* human epiderma growth factor receptor 2, *TOP2A* topoisomerase II alpha

**Supplementary Table 4** Univariate and multivariate analysis of variables correlated with DFS and OS in HR-negative patients.

| Variables | Univariate analysis | | | | Multivariate analysis | | | |
| --- | --- | --- | --- | --- | --- | --- | --- | --- |
|  | DFS | | OS | | DFS | | OS | |
|  | Hazard Ratio (95%CI) | *P* Value | Hazard Ratio (95%CI) | *P* Value | Hazard Ratio (95%CI) | *P* Value | Hazard Ratio (95%CI) | *P* Value |
| **Age, years** |  | 0.323 |  |  |  |  |  |  |
| <40 | Reference |  |  |  |  |  |  |  |
| 40-49 | 0.95 (0.18-5.21) | 0.956 |  |  |  |  |  |  |
| 50-59 | 1.30 (0.27-6.27) | 0.741 |  |  |  |  |  |  |
| 60-69 | 0.79 (0.11-5.64) | 0.817 |  |  |  |  |  |  |
| >=70 | 3.97 (0.66-23.84) | 0.132 |  |  |  |  |  |  |
| **Menopausal status** |  | 0.187 |  | 0.459 |  | 0.192 |  |  |
| Premenopausal | Reference |  | Reference |  | Reference |  |  |  |
| Postmenopausal | 2.00 (0.71-5.61) | 0.187 | 1.67 (0.43-6.47) | 0.459 | 1.99 (0.71-5.61) | 0.192 |  |  |
| **Histological type** |  | 0.79 |  | 0.694 |  |  |  |  |
| No special type | Reference |  | Reference |  |  |  |  |  |
| Other | 0.76 (0.10-5.72) | 0.79 | 1.51 (0.19-11.95) | 0.694 |  |  |  |  |
| **T stage** |  | 0.301 |  | 0.4 |  |  |  |  |
| T0/is/1 | Reference |  | Reference |  |  |  |  |  |
| T2 | 1.45 (0.54-3.86) | 0.459 | 1.72 (0.46-6.41) | 0.421 |  |  |  |  |
| T3 | 5.59 (0.70-44.78) | 0.105 | / | 0.991 |  |  |  |  |
| T4 | 3.49 (0.44-27.97) | 0.239 | 6.72 (0.75-60.50) | 0.09 |  |  |  |  |
| **N stage** |  | <0.001 |  | 0.008 |  | 0.005 |  | 0.016 |
| N0 | Reference |  | Reference |  | Reference |  | Reference |  |
| N1 | 1.60 (0.41-6.18) | 0.497 | 2.57 (0.43-15.41) | 0.3 | 1.21 (0.29-5.07) | 0.796 | 2.50 (0.40-15.43) | 0.325 |
| N2 | 2.88 (0.60-13.88) | 0.187 | 6.34 (1.06-37.99) | 0.043 | 1.92 (0.35-10.62) | 0.453 | 4.69 (0.76-29.04) | 0.097 |
| N3 | 13.32 (4.44-39.99) | <0.001 | 15.06 (3.02-75.05) | 0.001 | 8.40 (2.34-30.20) | 0.001 | 13.82 (2.73-69.90) | 0.001 |
| **HER2 status** |  | 0.82 |  | 0.88 |  |  |  |  |
| HER2-zero | Reference |  | Reference |  |  |  |  |  |
| HER2-low | 0.90 (0.35-2.27) | 0.82 | 1.10 (0.32-3.80) | 0.88 |  |  |  |  |
| **TOP2A** |  | 0.742 |  | 0.16 |  |  |  | 0.295 |
| <30% | Reference |  | Reference |  |  |  | Reference |  |
| 30%-60% | 0.70 (0.26-1.94) | 0.495 | 0.30 (0.08-1.21) | 0.091 |  |  | 0.35 (0.09-1.46) | 0.151 |
| >60% | 0.66 (0.17-2.55) | 0.545 | 0.26 (0.03-2.16) | 0.213 |  |  | 0.36 (0.04-3.29) | 0.368 |
| **Endocrine therapy** |  | 0.323 |  | 0.098 |  |  |  |  |
| No/Unknown | Reference |  | Reference |  |  |  |  |  |
| Yes | 2.10 (0.48-9.16) | 0.323 | 3.71 (0.79-17.52) | 0.098 |  |  |  |  |
| **Radiotherapy** |  | 0.02 |  | 0.194 |  | 0.265 |  |  |
| No/Unknown | Reference |  | Reference |  | Reference |  |  |  |
| Yes | 3.74 (1.23-11.36) | 0.02 | 2.45 (0.63-9.48) | 0.194 | 2.13 (0.56-8.06) | 0.265 |  |  |
| **Chemotherapy** |  | 0.617 |  | 0.542 |  |  |  |  |
| No/Unknown | Reference |  | Reference |  |  |  |  |  |
| Yes | 0.69 (0.16-3.00) | 0.617 | 22.83 (0.001-5.26E+5) | 0.542 |  |  |  |  |

*HR* hormone receptor, *DFS* Disease-free survival, *OS* Overall survival, *HER2* human epiderma growth factor receptor 2, *TOP2A* topoisomerase II alpha

**Supplementary Table 5** Univariate and multivariate analysis of variables correlated with OS in HR-positive patients within and after 5 years.

| Variables | Univariate analysis | | | | Multivariate analysis | | | |
| --- | --- | --- | --- | --- | --- | --- | --- | --- |
|  | OS (within 5 years) | | OS (after 5 years) | | OS (within 5 years) | | OS (after 5 years) | |
|  | Hazard Ratio (95%CI) | *P* Value | Hazard Ratio (95%CI) | *P* Value | Hazard Ratio (95%CI) | *P* Value | Hazard Ratio (95%CI) | *P* Value |
| **Age, years** |  | 0.967 |  | 0.236 |  |  |  |  |
| <40 | Reference |  | Reference |  |  |  |  |  |
| 40-49 | 0.76 (0.24-2.39) | 0.634 | 1.65 (0.19-14.14) | 0.647 |  |  |  |  |
| 50-59 | 1.00 (0.37-2.74) | 0.999 | 2.26 (0.26-19.33) | 0.457 |  |  |  |  |
| 60-69 | 0.83 (0.26-2.63) | 0.756 | 0.63 (0.04-10.12) | 0.746 |  |  |  |  |
| >=70 | 1.23 (0.31-4.81) | 0.767 | 5.95 (0.62-57.19) | 0.123 |  |  |  |  |
| **Menopausal status** |  | 0.434 |  | 0.538 |  |  |  |  |
| Premenopausal | Reference |  | Reference |  |  |  |  |  |
| Postmenopausal | 0.75 (0.36-1.56) | 0.434 | 1.38 (0.50-3.79) | 0.538 |  |  |  |  |
| **Histological type** |  | 0.102 |  | 0.985 |  | 0.123 |  |  |
| No special type | Reference |  | Reference |  | Reference |  |  |  |
| Invasive lobular | 4.88 (1.04-22.87) | 0.044 | / | 0.986 | 5.46 (1.07-27.87) | 0.041 |  |  |
| Other | 2.03 (0.47-8.87) | 0.345 | 0.84 (0.11-6.37) | 0.864 | 1.30 (0.29-5.87) | 0.733 |  |  |
| **Grade** |  | 0.464 |  | 0.406 |  |  |  |  |
| Grade I | Reference |  | Reference |  |  |  |  |  |
| Grade II |  | 0.918 | 2.03 (0.26-16.01) | 0.503 |  |  |  |  |
| Grade III |  | 0.919 | 4.46 (0.52-38.24) | 0.172 |  |  |  |  |
| **T stage** |  | 0.783 |  | 0.043 |  |  |  | 0.147 |
| T0/is/1 | Reference |  | Reference |  |  |  | Reference |  |
| T2 | 1.14 (0.53-2.42) | 0.739 | 1.27 (0.42-3.89) | 0.672 |  |  | 1.22 (0.40-3.74) | 0.729 |
| T3 | 0.41 (0.05-3.22) | 0.394 | 11.63 (2.47-54.82) | 0.002 |  |  | 8.66 (1.67-44.81) | 0.01 |
| T4 | 1.45 (0.19-11.17) | 0.723 | / | 0.992 |  |  | / | 0.992 |
| **N stage** |  | 0.284 |  | 0.39 |  |  |  |  |
| N0 | Reference |  | Reference |  |  |  |  |  |
| N1 | 2.92 (0.62-13.78) | 0.176 | 2.43 (0.74-7.97) | 0.142 |  |  |  |  |
| N2 | 3.13 (0.65-14.99) | 0.153 | 2.51 (0.49-12.95) | 0.271 |  |  |  |  |
| N3 | 4.36 (0.96-19.76) | 0.056 | 4.60 (0.89-23.73) | 0.068 |  |  |  |  |
| **HER2 status** |  | 0.662 |  | 0.862 |  |  |  |  |
| HER2-zero | Reference |  | Reference |  |  |  |  |  |
| HER2-low | 0.82 (0.33-2.03) | 0.662 | 0.89 (0.25-3.18) | 0.862 |  |  |  |  |
| **Ki-67** |  | 0.101 |  | 0.542 |  | 0.046 |  |  |
| <15% | Reference |  | Reference |  | Reference |  |  |  |
| 15%-30% | 1.50 (0.54-4.22) | 0.438 | 1.21 (0.34-4.32) | 0.764 | 1.11 (0.37-3.33) | 0.852 |  |  |
| >30% | 2.96 (1.01-8.66) | 0.048 | 2.02 (0.54-7.54) | 0.297 | 2.99 (0.97-9.21) | 0.056 |  |  |
| **TOP2A** |  | 0.85 |  | 0.218 |  |  |  |  |
| <30% | Reference |  | Reference |  |  |  |  |  |
| 30%-60% | 1.28 (0.55-3.02) | 0.569 | 2.61 (0.89-7.64) | 0.081 |  |  |  |  |
| **Endocrine therapy** |  | 0.811 |  | 0.064 |  |  |  | 0.208 |
| No/Unknown | Reference |  | Reference |  |  |  | Reference |  |
| Yes | 0.90 (0.38-2.13) | 0.811 | 0.30 (0.09-1.07) | 0.064 |  |  | 0.42 (0.11-1.62) | 0.208 |
| **Radiotherapy** |  | 0.996 |  | 0.397 |  |  |  |  |
| No/Unknown | Reference |  | Reference |  |  |  |  |  |
| Yes | 1.00 (0.46-2.20) | 0.996 | 1.55 (0.56-4.27) | 0.397 |  |  |  |  |
| **Chemotherapy** |  | 0.057 |  | 0.537 |  | 0.084 |  |  |
| No/Unknown | Reference |  | Reference |  | Reference |  |  |  |
| Yes | 2.31 (0.98-5.45) | 0.057 | 1.44 (0.46-4.51) | 0.537 | 2.28 (0.90-5.79) | 0.084 |  |  |

*HR* hormone receptor, *OS* Overall survival, *HER2* human epiderma growth factor receptor 2, *TOP2A* topoisomerase II alpha

**Supplementary Figure 1** DFS and OS in HER2-zero and HER2-low patients. (**A**) DFS in HER2-zero and HER2-low patients. (**B**) OS in HER2-zero and HER2-low patients. *DFS* Disease-free survival, *OS* Overall survival, *HER2* human epiderma growth factor receptor 2


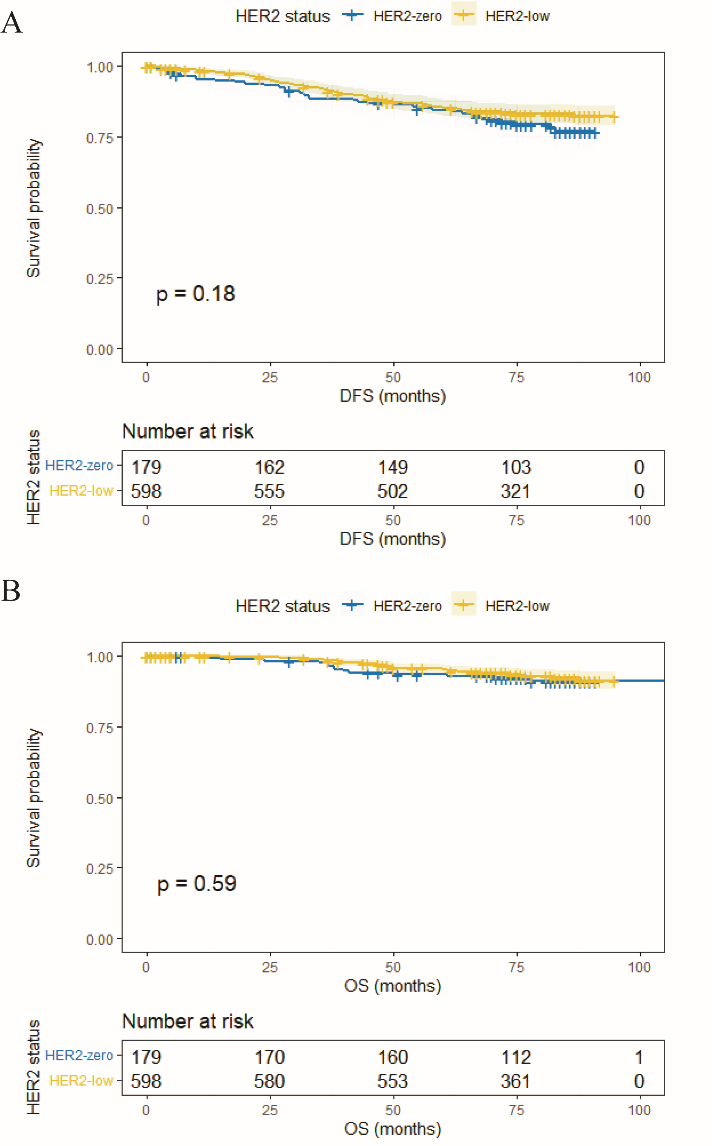

Supplement: Supplementary file 1 [file DataSheet_1.docx]
